# Some Phases of Quackery in Relation to Diseases of the Eye

**Published:** 1921-12

**Authors:** Cyril H. Walker

**Affiliations:** Ophthalmic Surgeon to the Bristol General Hospital; Surgeon to the Bristol Eye Hospital; Lecturer on Ophthalmology in the University of Bristol


The Bristol

Medico-Chirurgical Journal.

" Scire est nescire, nisi id me
Scire alius sciret.

DECEMBER, 1921.

### SOME PHASES OF QUACKERY IN RELATION TO DISEASES OF THE EYE.

The Presidential Address, delivered on October 12th, 1921, at the opening of the Forty-eighth Session of the Bristol Medico-Chirurgical Society.

BY

Cyril H. Walker, M.B., B.A. Cantab.,

*Ophthalmic Surgeon to the Bristol General Hospital ; Surgeon to the Bristol Eye Hospital ; Lecturer on Ophthalmology in the University of Bristol.*

In the first place I wish to thank you for the very great honour you have done me in electing me your President for this year. I feel that I am not worthy of holding so distinguished a position, and that it is to some extent a recognition of the importance of the branch of medicine with which my work has been associated. The first meeting of this Society that I ever attended, thirty years ago this week, was addressed from the presidential chair by that distinguished ophthalmologist Mr. Richardson Cross, whose work for the advancement of ophthalmology throughout the whole of the West of England is so well known to you all.

That I am elected to follow him and many other distinguished Presidents in occupying this chair is an honour which I assure you I appreciate very highly. I promise to conduct the business of these meetings to the best of my ability, but I fear that I shall have to ask your indulgence on many occasions, since the task for one who has practised only as a specialist is by no means an easy one.

Scarcely any branch of medicine has been more invaded by advertising charlatans than has ophthalmology, and I propose to call your attention this evening to some phases of quackery in relation to diseases of the eye. The term "Quack" or "Quack-salver" came into common use in the reign of Charles I., and refers of course to the noisy, reiterated chatter of the medicine vender at fairs. A quack doctor has been defined as "a boastful pretender to medical skill, an empiric, an ignorant practitioner." Quackery is, however, to some extent a relative term. Much of our practice is still founded on empiricism, and the empiric of to-day may quite possibly be regarded by the practitioners of a generation or two hence as having been guilty of quackery. The practice of bleeding patients before and after operation, as was customary a hundred years ago, might be denounced as quackery. Sir Wm. Blizard, the eminent surgeon, was operated upon for cataract at the age of 92. He was bled to 8 ounces on the evening of the operation. Lawrence in his *Venereal Diseases of the Eye* relates the case of a prize-fighter suffering from gonorrhœal ophthalmia who was bled to 152 ounces in four days, besides having 44 leeches applied. Pure empiricism is not necessarily quackery. As Dr. Gould of Philadelphia puts it, "a quack is a man more interested in himself than in his healing art; caring more for his patent than his patient." The doctor is not called upon to cure a disease but to treat a patient. Quackery to a great extent ignores the patient, and confines its attention to the disease, *i.e.* it unduly exalts the remedy.

During the Middle Ages the treatment of diseases of the eye was very largely in the hands of itinerant quacks, who went from fair to fair selling nostrums to the credulous, and couching cataracts when occasion offered. Now I think there can hardly be any more satisfactory operation in the whole realm of surgery than the successful removal of a cataract from a blind patient, but the operation of couching, that is depressing the opaque lens from the pupil into the lower part of the vitreous, though giving brilliant results for the moment, was often followed by glaucoma or other serious diseases of the eye, so that the itinerant quack had a great advantage over the regular practitioner, who had to abide the consequences of his handiwork, whereas the quack, after the fee was paid, need not see his patient again. Couching is still the routine treatment for cataract in India. These itinerant operators care little for the ultimate success of the operation, and investigations show that at least two-thirds of the cases are immediately or ultimately failures.

For thousands of years there seems to have been practically no advance in the treatment of cataract, until 1745, when Daviel in France began the operation of extraction of the lens.

It was about the time of James I. that those practising ophthalmology were first called "oculists." Most of them were quack-salvers, and the term oculist has in consequence never found much acceptance with members of the medical profession. Further, some of these oculists boasted of their entire ignorance of medicine and surgery, regarding such knowledge as confusing and detrimental to success.

Early in the eighteenth century quackery sprang into increased importance, and spread the world over, from China to Peru. Infallible remedies for all sorts of diseases were freely advertised. Oliver Goldsmith in his *Citizen of the World* observes, " There must be something strangely obstinate in an English patient who refuses so much health upon such easy terms."

Queen Anne, whose defective sight rendered her a god-send to quack oculists, revived the custom of " touching " for King's Evil, a procedure which a generation before was so frequently resorted to that it is recorded that Charles II. " touched " 92,000 of his subjects in 22 years, and of course took fees—" touch-pieces " as they were called—for doing so. It will be remembered that Dr. Johnson was taken to Queen Anne to be " touched." She was the last sovereign to assert a claim to the power of healing King's Evil. Herself a quack, and fond of anything mysterious, it is no wonder that she took an interest in quackery. She even went so far as to appoint as her oculists two charlatans. One was William Read, a tailor. In 1705 he received a knighthood, the ostensible reason being that he had " cured great numbers of seamen and soldiers gratis." The other quack was Roger Grant, a shoemaker, or as some say a tinker. He had lost one eye when serving as a soldier in the army of the Emperor of Germany. In spite of, or perhaps because of, this disability he set up as an oculist in Mouse Alley, Wapping. Addison alludes to him as being notorious for " putting out eyes with great success." In this irregular way began the now much-coveted and respected title of " Surgeon-oculist to the King."

About the middle of the eighteenth century there were many other "ambulant " practitioners who toured the country accompanied by all the apparatus of shameless advertisement (including " monkeys " we are told), couching cataracts and selling infallible salves and eye-washes. One of these preparations is still popular, Singleton's Golden Eye

Ointment. It was advertised as being able to cure all diseases of the eyes. This ointment, which is 7 per cent. red oxide of mercury, was invented, or, as some put it, discovered close upon 300 years ago, but most of these nostrums have passed away with their unscrupulous inventors.

Supreme amongst quacks of this period was "The Chevalier" Taylor, a full account of whose life occurs in the Royal London Ophthalmic Hospital Reports for 1915. He was a qualified surgeon, a man of immense energy and ability, and had travelled all over Europe. He was appointed "Ophthalmiator Pontifical" by Pope Benedict XIV., and was oculist to the Kings of England, Denmark, Poland, Norway and Sweden. His entry into towns which he honoured with his presence was not exactly by stealth. In what he calls "the crisis of his grandeur" he travelled "with no less than two coaches and six, above ten servants in livery, besides gentlemen, my companions, in my own pay." It is said that his coach was painted over with eyes, and bore the motto, "Qui visum dat, vitam dat." Leaflets, placards, and advertisements in the public press heralded his arrival. A sort of reception in the biggest hall in the town was arranged, and the gentry were invited every morning to see his method of restoring sight. He was frequently in conflict with his professional brethren. It was he who operated on Handel for cataract, "but on drawing the curtain we found the bottom defective." Though an unparalleled liar, he was by many tolerated as an amusing rascal.

Compared with the Chevalier Taylor present-day quacks may seem tame and dull. For anything like his audacity one has to go to America. The "means to do ill deeds" is sometimes the bogus qualification or degree, an useful asset to the quack. "Carnegie University, of Wilmington,

Delaware," was one of the bodies granting such degrees. It was successfully exposed by the American Medical Association. It appears that this University was run by a "Board of Regents," who began operations by conferring its degrees upon themselves. "What is the use of running a diploma mill unless you can furnish yourself with any title that may strike your fancy?" So five out of the eight Regents modestly conferred upon themselves the degree of M.D. A wily member of the staff of the journal of the American Medical Association wrote to Carnegie University applying for a degree, stating that he wished to practise as a bone-setter in England. He received in reply an examination paper to answer. Of course his answers were suitably faked. One of the questions was, "What is Keratitis? Give course and treatment." He answered, "Keratitis is an inflammation of the eye. It should be treated by manipulating the nerves and muscles of the neck, and by adjusting the vertebra of the neck." The treatment for Iritis was to be the same as for Keratitis. This was the only question on eye disease. I quote it to show that the long string of questions was equally applicable to the quack oculist or the bone-setter. The Board of Regents wrote that the candidate had passed "a very satisfactory examination," and that the diploma he wanted would be forwarded to him on receipt of fifty dollars. It is satisfactory to record that the President and Secretary of the Carnegie University were shortly afterwards imprisoned for fraud. The unsuspecting public are easily taken in by any self-styled oculist who cares to avail himself of such bogus diplomas.

In the States there were firms who made a business of collecting or even manufacturing laudatory letters and glowing testimonials in proof of the efficacy of any sort of patent medicine, and of the ability of any impostor who perambulated the country defrauding the ignorant.

The druggist in France is, I believe, forbidden to sell secret remedies or even keep them on his premises, and yet quack remedies flourish and abound in France. I shall never forget the well-known ophthalmic surgeon Dr. Darier of Paris recounting at a meeting in Oxford his experience of a firm of ophthalmic quacks who pitched their stumps in Paris. It was about the time when Bier's hyperæmic treatment was in vogue, and these rogues seem to have borrowed their notion of curing errors of refraction from Bier's suction apparatus. Darier consulted them one evening. The operator in a casual way picked up a sheet of paper, made two pencil dots on it and asked Dr. Darier what was the greatest distance at which he could distinguish the marks as being two separate dots. The limit was about one metre. An elaborate and magnificent suction apparatus, all polished and glittering, was applied to his orbital region, and in a most impressive manner a pumping movement was conducted, creating a slight vacuum. The test dots were again exhibited, but there was no material improvement. A second or third application of the instrument was then tried, after which Darier found that he could discern the dots at nearly two metres. All might have gone well had not the performer moved away, apparently to make some record of the result. Darier had the curiosity to pick up some sheets of paper lying on the table. On the back of each sheet he found two very conspicuous dots. He then looked at the sheet with which he had been tested, and found two small dots on the front and two much larger and more conspicuous ones on the back, the ones in fact which he had just been able to see at the greater distance. The inference was irresistible. As Dr. Darier quaintly put it, "His confidence was much shaken." I fancy the operators were next heard of in America, but of their subsequent history nothing can be ascertained.

The epidemic of quackery spread in Germany just as everywhere else, and was very active shortly before the great war. Emil Schlegel of Tübingen in 1887 discovered, or pretended he discovered, that the human iris, by its tints, fleckings and colours denotes the part of the body injured or diseased; that its spots, according to location, mean wounds of the ear or the skin, a syphilitic tumour, lung disease, prolapse of the uterus, etc. How anyone could have tolerated such humbug is a thing imagination boggles at. Palmistry is credibility itself by comparison.

Prior to the war there was in Wiesbaden a clinic which attracted large numbers of patients from all parts of the world, amongst whom of course were many hopeless cases. Amid much that was scientific and genuine there often seemed to run a vein of quackery. The seemingly hopeless case usually had wet pack and mercurial inunction. Several patients who have been under my own care resorted to this clinic. One man was suffering from a tumour of the pituitary body, another from alcoholic amblyopia. Both alike were subjected to wet pack and inunction. The one with amblyopia was at Wiesbaden when war was declared and had great difficulty in returning to England. The fact that this poor man wore blue glasses and spoke no German placed him at once under the gravest suspicion of being a spy. It is easy at any time to misinterpret evidence and to jump to wrong conclusions. You will recollect that a German newspaper described Holloway as "a town near London noted for its pills."

There have been in England from time to time, even in recent years, gross examples of ophthalmic quackery. About thirty-five years ago a party of Indian oculists travelled up and down the country professing to cure cataracts and other eye diseases. "Omne ignotum pro magnifico." These orientals, with no qualifications for the art to which they made pretension other than a dusky skin and a smattering of English, made easy dupes of the ignorant, but eventually they got into trouble in Kent. A poor woman took her child aged 10 to these vagabonds. She was informed that each of the child's eyes contained a worm. After three weeks' treatment, and a payment by the mother of £2 4s. 0d., they alleged that the worms had been extracted. The quacks were prosecuted, convicted and imprisoned. Sporadic cases similar to this crop up from time to time. Only a few years ago a blacksmith in South Wales was exhibiting great power in curing diseases of the eye. I believe his services are still in request, but I have been unable to find any recent records of his activities.

In England, and I believe on the Continent and in America as well, a good deal of quackery is associated with the prescribing of spectacles by opticians. Sometimes it is the itinerant eyesight expert who, at immense personal inconvenience, and on the earnest solicitation of countless sufferers, has decided to sacrifice his own interests and those of his clients in London, and to pay a flying visit to some provincial town, where he confidently expects he will receive an enthusiastic welcome. Paragraphs to this effect appear in the local press, and leaflets are distributed broadcast in the streets. These claim, with conspicuous lack of modesty, that there is no defect of sight which Mr. Blank does not remedy in the most effective manner. Sometimes these manifestoes do not say exactly what they mean. One warns the *in*credulous public against unscrupulous imitators; another states, ambiguously enough, that "unless there is a reasonable hope of the patient being benefited he is at once told so; but should the case present any diminution of hope, it is undertaken, and in almost every instance successfully so."

This is not the time or place to enter into a discussion of the pros and cons of granting licences to opticians. The Spectacle-makers Company and other bodies have instituted examinations in sight-testing, and it must be freely admitted that on the whole the public has greatly benefited by the improved knowledge of the subject which most opticians now possess. The public, however, have not yet grasped the fact that the oculist of to-day is a fully qualified doctor, while they are misled by a string of letters after an optician's name and regard him as having a medical qualification. Most opticians, no doubt, are competent and honest ; at the same time there is a very great temptation for them to supply spectacles of some sort even when they afford no help, otherwise the optician can get no recompense for the time he has expended on his customer. Sometimes more spectacles than are really necessary, or even desirable, are supplied. A glaring example of this kind of fraud came under my notice some years ago. A newly-married man, with more money than sense, consulted (that is the term) a West End optician, who ordered him two pairs of spectacles —gold frames, toric lenses, astigmatic correction, and all the rest of it—and charged 10 guineas, which was cheerfully paid. A few days later he received a letter from the optician inviting him to purchase a complete outfit of spectacles. There were glasses for the tropics, others for the Alps, glasses for billiards, for fishing, and for tying flies, shot-proof glasses, glasses for evening dress, and glasses for reading. Wherever possible there was a duplicate pair in pince-nez frames. The whole packed in a polished mahogany box with lock and key for 100 guineas. Deduct the 10 guineas already paid, and say £90 cash down. Fortunately, before closing with this comprehensive offer the gentleman was prevailed upon by his wife to consult an ophthalmic surgeon, who found that only one pair of glasses was needed.

Half a century ago some of the best spectacle lenses were made of quartz crystal, and opticians fostered the impression that wearing these "pebbles," as they were called, presented some great advantage. For a long time now the popularity of pebbles has waned, partly no doubt owing to the wearer being unable to detect the difference between quartz and glass, and so suspecting that though he had paid for pebbles he had after all only got glass. Recently the rage has been for lenses made according to the formula of Sir William Crookes. This glass was designed by him a good many years ago to protect the eyes of bottle makers and others working at furnaces from the excess of heat rays and ultra-violet rays. Half the opticians in the land are now advocating the use of Crookes' lenses or some variant of his original formula. A good deal of quackery results. The normal eye does not need shielding from a normal amount of ultra-violet rays, and there is no real evidence in favour of the indiscriminate use of Crookes' glass.

For many years I have known of an unqualified practitioner who claims the possession of a secret method of curing visual defects, which has been handed down from generation to generation. Cataract, leucoma, even optic atrophy is not altogether beyond the reach of his remedies. I have seen several patients who have come under the spell of this charlatan. His leading line in all difficult cases seems to be the supplying of excessively strong lenses. A man with 12 dioptres of myopia and an advanced degree of cataract was ordered to wear a +8 sph. for constant wear, and to practise reading with a +14 sph. lens as a cure. His reading distance with this lens was about two inches. He found the treatment caused so much discomfort that after a month or two he had to abandon the cure.

The optician in difficult cases is at a very great disadvantage. A mydriatic is often absolutely necessary in order that refractive error may be accurately estimated, and the risk of glaucoma as the result of a mydriatic is so very definite that no unqualified practitioner should ever be allowed to use it. This risk has no doubt been recognised at last by the quack, and it is many years since I have seen a patient who was using "anti-cataract oil." This preparation contained belladonna in some form, and must have been responsible for very many cases of glaucoma. There are still, however, enterprising spirits who advertise something of the kind, though possibly not containing any belladonna. Only a few days ago I saw a farmer of 85 who for ten years had paid periodical visits (not to mention fees) to a quack who supplied him with drops intended to disperse an already mature cataract.

In 1910, at the instance of the General Medical Council, a Blue Book was published containing a great deal of information as to the prevalence of quackery and the harm it was doing to the nation. The market place of every country town is systematically visited by ignorant medicine venders who defraud the poor. The evidence collected shows that one of the reasons why ophthalmic quackery is so common is that the facilities for proper advice and treatment are inadequate or the cost of them beyond the means of the poor. Comparatively few doctors, it says, are qualified to do ophthalmic work or can afford the time for it. The result of this is that the public continue to think that defective sight is not a matter about which they need consult the family doctor, and so many of them go instead to the chemist or the optician.

Most of you know that there is a body called the "Council of British Ophthalmologists" which was instituted some three and a half years ago. Amongst various matters that have come under their consideration is the question of sight-testing by opticians. Their report on this matter has not yet been published, but it appears that for the present they recommend that no active steps be taken. Indeed, it seems obvious that a certain amount of testing for spectacles cannot be objected to. Why should not a presbyope of 50 buy a pair of spectacles without consulting a doctor, just as he would buy a pocket magnifying glass, or any of the appliances which mitigate the disabilities of advancing years. Presbyopia is not yet a notifiable disease, in fact it is hardly a disease at all. Neither the public nor the profession is likely to suffer much at the hands of the sight-testing optician, provided he knows his trade and is honest. But the possibility of his overlooking glaucoma, optic neuritis, or other eye diseases is considerable. The public should be warned of this, and have the opportunity of getting proper advice at a cost that is within their means. A State medical service should ensure the provision of a sufficient number of well-staffed and properly equipped ophthalmic clinics within easy reach both of town and country dwellers. This is already being done for children in elementary schools, and the parent may reasonably claim the same advantages as his children enjoy. Provision of these clinics will involve some increase in the number of trained ophthalmologists, but the numbers at present entering the medical profession are probably sufficient to meet the demand, and the various diplomas in ophthalmology which have recently been instituted in association with schools of ophthalmology are a great advance on the opportunities existing thirty years ago.

Lastly, the profession as a whole, particularly those in general practice, whether panel practice or not, must actively help in the suppression of quackery. Restrictive legislation may do a little towards this end, but it cannot do much. Quackery is a hundred-headed hydra, and cannot even be muzzled, let alone choked, by making it illegal. The family doctor can help by showing up examples of quackery, and by urging the authorities to provide the facilities I have suggested wherever he finds them lacking. He should, if possible, keep in touch with the nearest eye department or clinic, either by visiting the institution, or at least by writing or telephoning about the patients he sends there for consultation. He will thus maintain an interest in ophthalmology, and will learn better its possibilities and limitations, and thus be enabled to decide when to persuade his patients to seek special advice, rather than allowing them to drift to the optician or the quack. By doing this he will more than ever deserve to be regarded as their guide, philosopher and friend.

